# Supplementary material for: Association between oral microbiome and seven types of cancers in East Asian population: a two-sample Mendelian randomization analysis
Source: Front Mol Biosci. 2023 Nov 21;10:1327893. doi: 10.3389/fmolb.2023.1327893 (PMC10702768; doi:10.3389/fmolb.2023.1327893)
Supplement: Supplementary file 1 [file DataSheet1.ZIP › Supplementary Materials/MR plots for tongue/tongue═╝/Pancreatic cancer/╥╚╧┘░⌐_╔╕╤í╜ß╣√/pheno.1818_to_pancreatic cancer_forest.pdf]

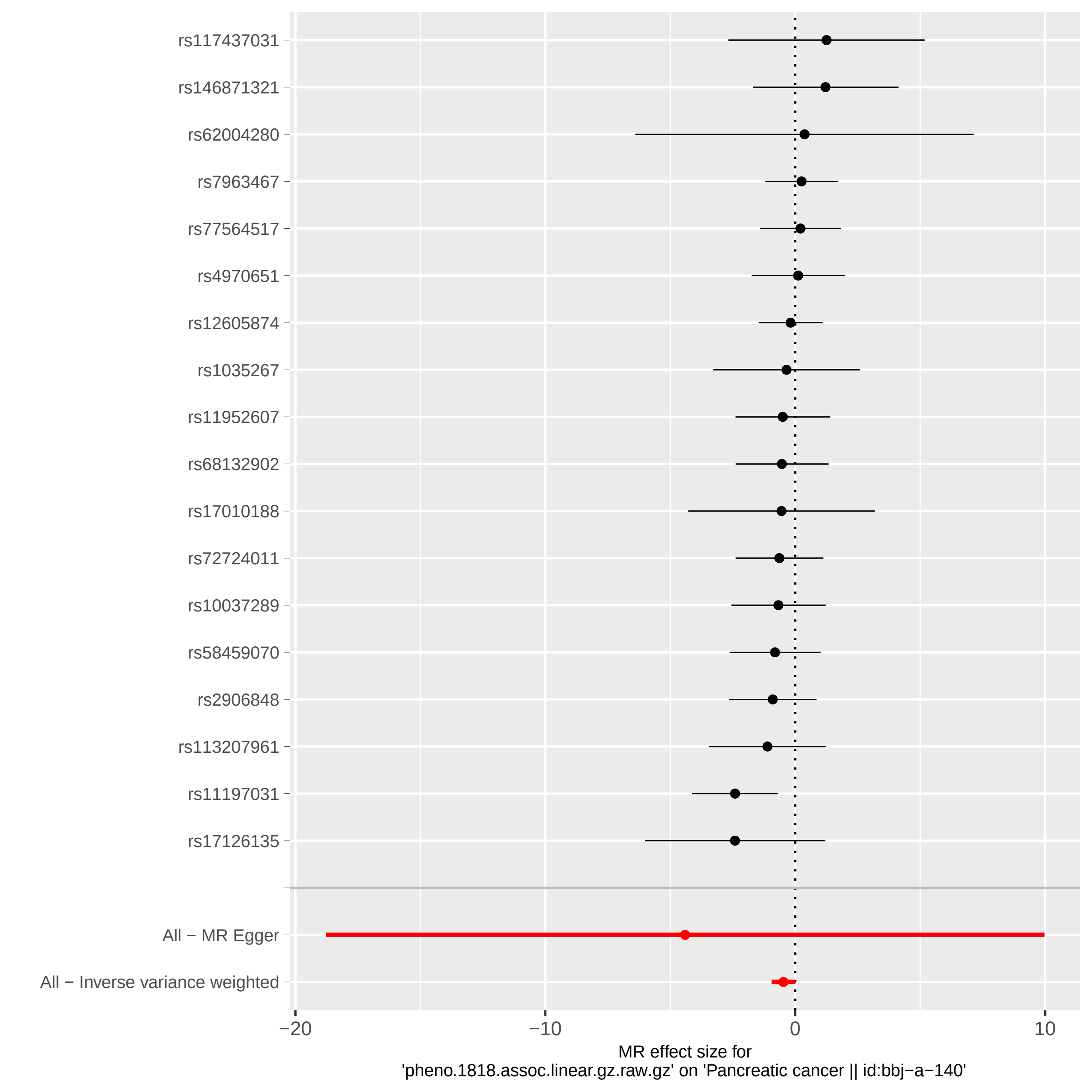

rs117437031
rs146871321
rs62004280
rs7963467
rs77564517
rs4970651
rs12605874
rs1035267
rs11952607
rs68132902
rs17010188
rs72724011
rs10037289
rs58459070
rs2906848
rs113207961
rs11197031
rs17126135
All − MR Egger
All − Inverse variance weighted
−20
−10
0
10
MR effect size for
'pheno.1818.assoc.linear.gz.raw.gz' on 'Pancreatic cancer || id:bbj−a−140'
